# The Trend in Industry Payments During the COVID-19 Pandemic Among Gastroenterologists and Hepatologists in the United States

**DOI:** 10.7759/cureus.32711

**Published:** 2022-12-19

**Authors:** Anju Murayama, Sae Kamamoto, Hiroaki Saito, Tetsuya Tanimoto, Akihiko Ozaki

**Affiliations:** 1 School of Medicine, Tohoku University, Sendai, JPN; 2 Faculty of Medicine, Hamamatsu University School of Medicine, Hamamatsu, JPN; 3 Department of Internal Medicine, Soma Central Hospital, Soma, JPN; 4 Department of Internal Medicine, Navitas Clinic, Tokyo, JPN; 5 Department of Breast and Thyroid Surgery, Jyoban Hospital, Tokiwa Foundation, Iwaki, JPN

**Keywords:** industry payment, open payments database, conflict of interest, covid-19, gastroenterologists and hepatologists

## Abstract

Background

Although the sudden coronavirus disease 2019 (COVID-19) pandemic would have significantly influenced financial relationships between the healthcare industry and gastroenterologists and hepatologists, little is known about the trend in financial relations in the United States. This study, thus, aimed to examine the trends in industry payments made to gastroenterologists and hepatologists during the COVID-19 pandemic.

Materials and methods

Using the Open Payments Database between 2013 and 2021, we evaluated trends in financial relationships between the healthcare industry and gastroenterologists and hepatologists in the United States. Trends in general payments during the COVID-19 pandemic were evaluated by interrupted time series analysis with monthly and yearly payments at the physician level.

Results

A total of 16,808 or 89.4% of all active gastroenterologists received general payments totaling $393,823,094 from the pharmaceutical and medical device companies between 2013 and 2021. The payment per gastroenterologist and the number of gastroenterologists receiving payments decreased by 70.9% (95% CI: -73.4% - -68.1%, p<0.001) and by 51.5% (95%CI: -52.2% - -50.7%, p<0.001) due to the onset of the COVID-19 pandemic, respectively. However, both payments and the number of physicians with payments have recovered monthly since the COVID-19 pandemic, with relative monthly change rates of 4.1% (95% CI: 3.5% ‒ 4.7%, p<0.001) and 3.2% (95%CI: 3.1% ‒ 3.2%, p<0.001). Additionally, the general payments per gastroenterologist significantly decreased by 2.5% (95%CI: -3.9% - -1.1%, p<0.001) each year before the COVID-19 pandemic, while there was a very small change in the number of gastroenterologists with payments.

Conclusions

The industry payments to gastroenterologists and hepatologists significantly decreased due to the COVID-19 pandemic, but the payments have recovered right after the pandemic in the United States.

## Introduction

A widespread and sometimes strong financial relationship between the healthcare industry and gastroenterologists and hepatologists has been repeatedly reported in the United States [1-3]. Gastroenterology and hepatology is one of the specialties with the largest number of physicians and demand in the United States. One recent study found 87.7% of gastroenterologists received one or more non-research payments between 2014 and 2020 [4]. The study reported that total non-research payments including food and beverage, speaking fees, consulting fees, and travel and accommodation fees, increased by 11.4% each year between 2014 and 2016. Then, total payments decreased by 5.8% from 2016 to 2019. 

The restriction on people’s social activities due to the sudden and urgent onset of the coronavirus disease 2019 (COVID-19) pandemic might have reduced opportunities for promotional activities by the industries and consequently financial relationships with industries among the gastroenterologists in the United States [5]. A recent study reported that the non-research payments from the healthcare industry to physicians decreased by 48.4% in 2020 [5]. However, no study has assessed the trend in industry payments specifically among gastroenterologists and hepatologists during the COVID-19 pandemic in the United States.

## Materials and methods

Study design and study participants

Using the public payment database, the Open Payment Database, this cross-sectional study examined the trend in general payments made by the healthcare industries to all active gastroenterologists and hepatologists between August 2013 and December 2021 in the United States.

Data collection

The general payments to gastroenterologists and hepatologists were extracted by following steps. First, we sorted all physicians who specialized in gastroenterology and hepatology (“Allopathic & Osteopathic Physicians|Internal Medicine|Gastroenterology” “Allopathic & Osteopathic Physicians|Internal Medicine|Hepatology” and “Allopathic & Osteopathic Physicians|Petiatrics|Pediatric Gastroenterology”) in the National Plan and Provider Enumeration System (NPPES) database. Second, we extracted the profile data of all gastroenterologists and hepatologists who were continuously active between August 2013 and December 2021 from the NPPES database. Finally, we extracted all general payments between August 2013 and December 2021 from the Open Payments Database by matching the physicians' National Provider Identifier (NPI) numbers, which we extracted from the NPPES database. The Open Payments Database first released the payment data in August 2013 and the latest payment data was in 2021. Therefore, we used all the available payment data for this analysis. The general payments were made to physicians for non-research and non-ownership purposes, including meals, lecturing compensations, consulting fees, travel and accommodation fees, and education fees. 

Data analyses

Descriptive analyses were conducted for payment data each year and overall. To evaluate the trend in payments, the interrupted time series analysis with the panel-data of monthly payments per physician was performed using the population-averaged generalized estimating equation models (GEE) [6]. The yearly trends in payment per gastroenterologist and the number of gastroenterologists with payments were also evaluated by the population-averaged GEE models, and the yearly trends in total payment values and contracts were evaluated by the linear regression models. As the payments were not normally distributed, the negative binomial GEE model for payments per physician and linear log-linked GEE model with the Poisson distribution for the number of physicians with payments were employed, as we noted previously [7-9]. As the national emergency was declared on March 13, 2020, in the United States, we considered the period before and after March 2020 as before the COVID-19 pandemic and during the pandemic. Predictor variables included the onset of the COVID-19 pandemic, month of payment, month since the COVID-19 pandemic, and monthly variables to adjust the monthly trend in payment patterns. Additionally, the USD (United States Dollar) inflation rate was adjusted by dividing the inflation rate by the consumer price index for each month, relative to August 2013. All analyses were performed using Microsoft Excel for Microsoft 365 MSO, version 2202 (Microsoft Corporation, Redmond, Washington, United States), and Stata Statistical Software: Release 17 (2021; StataCorp LLC, College Station, Texas, United States). P-values were statistically significant at 0.05.

Ethical clearance

The Ethics Committee of the Medical Governance Research Institute, Tokyo Japan, approved this study. Informed consent from the participants was waived by the Ethics Committee of the Medical Governance Research Institute, as this study was a cross-sectional study of the public database.

## Results

**Figure 1:**
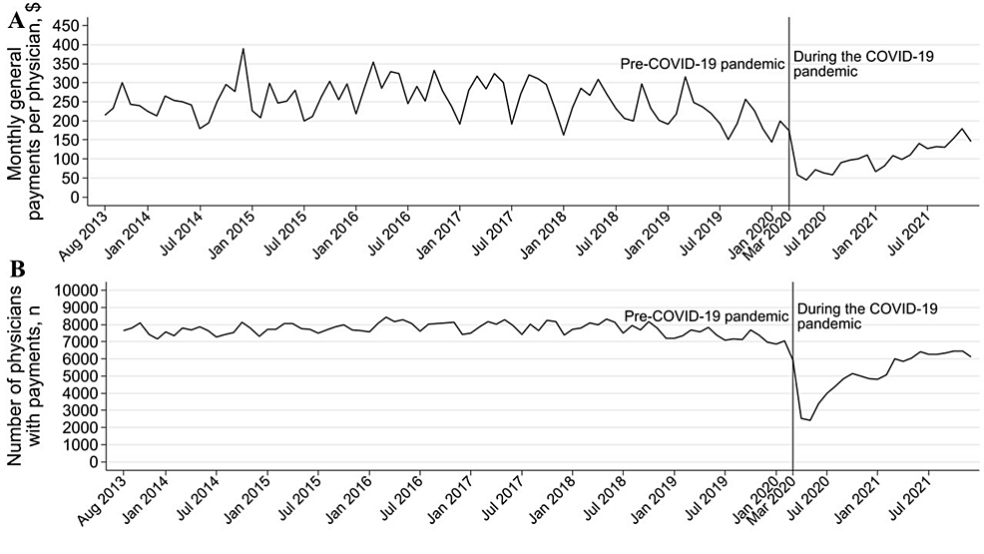
Monthly general payments per physician and number of gastroenterologists and hepatologists with payments before and during the COVID-19 pandemic in the United States A: Trend in the monthly general payments per gastroenterologist and hepatologist before and during the COVID-19 pandemic; B: Trend in the monthly number of gastroenterologists and hepatologists receiving general payments before and during the COVID-19 pandemic COVID-19: coronavirus disease 2019

A total of 16,808 (89.4% of all active gastroenterologists and hepatologists) received general payments totaling $393,823,094 and 3,061,232 contracts from pharmaceutical and medical device companies between 2013 and 2021 (Table 1). Median monthly payments per gastroenterologist receiving payments were $62.46 (interquartile range (IQR): $26.4-$127.76) before the COVID-19 pandemic and $53.04 (IQR: $23.29-$116.915) during the pandemic. The payments per gastroenterologist and number of gastroenterologists receiving payments declined by 70.9% (95% confidence interval (CI): -73.4% - -68.1%, p<0.001) and by 51.5% (95%CI: -52.2% - -50.7%, p<0.001) at the onset of the COVID-19 pandemic, respectively (Figure 1 and Table 2). However, both payments and the number of physicians with payments have recovered monthly since the COVID-19 pandemic, with relative monthly change rates of 4.1% (95%CI: 3.5%‒4.7%, p<0.001) and 3.2% (95%CI: 3.1%‒3.2%, p<0.001). We found small decreasing trends by 0.2% (95%CI: -0.3% - -0.1%, p<0.001) in monthly payments per gastroenterologist and by 0.03% (95%CI: -0.05 - -0.01, p<0.001) in the number of gastroenterologists receiving payments before the COVID-19 pandemic.

**Table 1:** Summary of annual general payments made to gastroenterologists and hepatologists between 2013 and 2021 in the United States *P<0.05, **P<0.01, P<0.001 ^a ^Only physicians receiving payments each year were included for average and median calculations; ^b ^The payments in 2013 were excluded from the trend analysis, as the payments in 2013 were partially disclosed between August and December. Relative annual changes in the variables were reported as a relative percentage; ^c ^Relative differences of each variable between 2020 and 2021 compared to those averaging between 2014 and 2019 were reported as a relative percentage; ^d ^Relative annual changes in the variables were reported as a relative percentage CI: confidence interval; IQR: interquartile range; SD: standard deviation

Variables	Year									Relative change rate (95% CI), %			Overall
	2013	2014	2015	2016	2017	2018	2019	2020	2021	Annual change between 2014-2019^b^	2014-2019 vs 2020-2021^c^	Annual change between 2020-2021^d^	
Total payments, $	20,694,039.71	51,560,718.78	51,878,375.68	59,170,058.27	58,395,718.32	52,299,128.91	48,310,907.09	22,584,510.73	28,929,636.81				393,823,094
Number of physicians with payments, n	11,175 (59.4)	12,686 (67.5)	13,339 (70.9)	13,400 (71.3)	13,280 (70.6)	13,164 (70.0)	12,592 (67.0)	10,335 (55.0)	10,603 (56.4)	-0.2 (-0.4 – -0.1)*	-22.5 (-23.9 – -21.1)***	2.8 (1.7 – 4.0)***	16,808
Payments per phyisician, $													
Median (IQR)	226 (94–532)	456 (149–1,197)	410 (125–1,113)	469 (133–1,232)	441 (130–1,175)	455 (133–1,178)	415 (125–1,128)	227 (72–656)	371 (102–1,135)	-2.5 (-3.9 – -1.1)**	-65.4 (-68.6 – -61.8)***	25.5 (18.7 – 32.7)***	2,752 (706–8,182)
Average (SD)	1,852 (8,098)	4,064 (21,910)	3,889 (19,080)	4,416 (19,647)	4,397 (19,450)	3,973 (17,921)	3,837 (17,058)	2,185 (9,745)	2,728 (10,960)				23,431 (108,181)
Range	0.96–272,956	2.67–1,187,662	0.03–476,283	0.03–409,986	0.03–447,471	0.78–494,666	0.78–463,529	0.83–293,052	0.48–237,568				0.03–3.013,848

**Table 2:** Relative monthly change rate in payments per gastroenterologist and number of gastroenterologists with payments before and during the COVID-19 pandemic *p<0.05, **p<0.01, ***p<0.001 Relative monthly change in each variable was reported as a relative percentage COVID-19: coronavirus disease 2019

Variables	Relative monthly change rates, % (95% confidence interval)	
	Payment per gastroenterologist	Number of gastroenterologists with payments
Before the COVID-19	-0.2 (-0.3 ‒ -0.1)***	-0.03 (-0.05 - -0.01)***
The COVID-19 pandemic onset	-70.9 (-73.4 ‒ -68.1)***	-51.5 (-52.2 - -50.7)***
During the COVID-19 pandemic	4.1 (3.5 ‒ 4.7)***	3.2 (3.1 - 3.2)***
Month		
January	Ref.	Ref.
February	21.3 (15.7 ‒ 27.2)***	2.0 (1.5 - 2.6)***
March	79.2 (70.2 ‒ 88.7)***	12.6 (12.0 - 13.2)***
April	32.3 (25.6 ‒ 39.4)***	4.1 (3.5 - 4.6)***
May	37.6 (30.6 ‒ 44.9)***	5.2 (4.6 - 5.8)***
June	42.3 (34.9 ‒ 50.1)***	4.6 (4.0 - 5.2)***
July	11.5 (5.6 ‒ 17.8)***	0.3 (-0.2 - 0.9)
August	14.4 (8.9 ‒ 20.2)***	3.6 (3.0 - 4.1)***
September	29.0 (22.2 - 36.2)***	3.7 (3.1 - 4.2)***
October	54.3 (46.7 - 62.2)***	8.0 (7.4 - 8.5)***
November	39.6 (32.6 - 47.0)***	4.1 (3.5 - 4.7)***
December	34.2 (21.6 - 48.2)***	-2.4 (-2.9 - -1.9)***

Regarding the analysis based on yearly payments, the number of gastroenterologists with payments did not change by year before the COVID-19 pandemic, ranging from 12,592 (67.0% of all gastroenterologists and hepatologists) in 2019 to 13,400 (71.3%) in 2016 (Table 1). The payments per gastroenterologist decreased from $469 (IQR: $133-$1,232) in 2016 to $415 (IQR: $125-$1,128) in 2019, with a relative annual change rate of -2.5% (95%CI: -3.9% - -1.1%, p=0.001). In 2020, 10,335 (55.0%) gastroenterologists and hepatologists received a total of $22,584,511 in general payments. The median per-physician payments were $227 (IQR: $72-$656), while average payments were $2,185 (standard deviation: $9,745) in 2020. The annual payments per gastroenterologist and number of gastroenterologists declined by 65.4% (95%CI: -68.6% - -61.8%, p<0.001) and by 22.5% (95%CI: -23.9% - -21.1%, p<0.001) in 2020, compared to those between 2014 and 2019, respectively. Additionally, 10,603 (56.4%) gastroenterologists and hepatologists received one or more general payments with $371 (IQR: $102-$1,135) in median in 2021. The per-physician payments and the number of gastroenterologists receiving general payments increased by 25.5% (95%CI: 18.7% - 32.7%, p<0.001) and 2.8% (95%CI: 1.7% - 4.0%, p<0.001), respectively (Table 1). These findings were consistent with the findings from the monthly analysis.

## Discussion

This analysis of monthly payment trends shows that the COVID-19 pandemic in the United States significantly contributed to the sudden decrease in general payments to gastroenterologists and hepatologists by less than half, in both payment amounts and the number of recipients. The decrease in non-research payments from the healthcare industry to healthcare professionals during the COVID-19 pandemic is described in several specialties. Inoue et al. found that the total general payments to physicians in 2020 reduced by 40-44% compared to that in 2018 and 2019 [4]. The non-research payments per internal medicine physician were reduced by 45.8% during the COVID-19 pandemic. Additionally, per-physician payments to allergists and clinical immunologists decreased by 52.6% and the number of allergists and clinical immunologists receiving payments also decreased by 36.9% due to the COVID-19 pandemic [8]. The number of infectious diseases physicians and per-physician payments to infectious diseases physicians also decreased by 58.6% and 54.4% during the COVID-19 pandemic, respectively [7]. This study adds to the evidence that physician-industry financial relationships were also restricted in the field of gastroenterology and hepatology due to the pandemic.

However, we also observed that there were increasing trends in payments and the number of gastroenterologists receiving payments right after the onset of the COVID-19 pandemic in the United States. Several studies reported that while the payments for meals and travel remained low, speaking compensations and consulting payments were less influenced and have recovered after the pandemic [4,8]. The reduction in non-research payments by industry is therefore likely to be temporary due to the unexpected and urgent COVID-19 pandemic and could recover to the same extent as before the pandemic.

Before the pandemic, Marshall et al. reported that the number of physicians accepting payments declined every year, as the percentage of all United State physicians receiving payments decreased by 13.8% overall and by 3.5% each year between 2014 and 2018 [9]. This decreasing trend was also observed in other specialties such as oncology, with a 4.9% annual decrease in the number of oncologists with payments [10]. However, our study elucidated that such a decrease in the number of physicians accepting payments was not observed among gastroenterologists and hepatologists between 2014 and 2019. Meanwhile, there was a decreasing trend in payment amounts between 2014 and 2019, possibly indicating a shift in companies' financing activities from non-research payments to research payments [11].

Owing to the Open Payments program inception, numerous studies demonstrated the non-research payments to physicians from the healthcare industry increased physicians’ prescriptions of brand-name drugs over generic alternatives and increased healthcare costs [12]. The drugs intensively promoted by the healthcare industry were sometimes lower in safety and efficacy, leading to low-quality prescriptions in the United States [12-15]. Although most physicians assume that industry payments and small gifts do not influence their clinical practice [16,17], accumulating evidence shows the financial interaction between physicians and the healthcare industry negatively influences patient care. Ideally, all physicians, including gastroenterologists and hepatologists, should decline to accept the non-research payments including meals and small gifts from the healthcare industry.

This study included several limitations. First, this study only included general payments. Second, the study included only gastroenterologists and hepatologists receiving general payments. Third, although the physicians can review and dispute the possible inaccuracies in the payment database listed in the Open Payments Database, only a small number of physicians disputed the payment data [18,19]. Therefore, there were possible inaccuracies in the Open Payments Database. However, the inclusion of all gastroenterologists with payments could evaluate the whole magnitude and trend in non-research payments from pharmaceutical and medical device companies in the United States.

## Conclusions

Non-research payments to gastroenterologists and hepatologists from the healthcare industry decreased by more than half due to the COVID-19 pandemic in the United States. However, the recovering trends in the non-research industry payments right after the pandemic indicates the sudden decrease in the payments was temporal.
